# Policy nudges toward medicalizing death and their impact on planetary health

**DOI:** 10.1177/08404704251348813

**Published:** 2025-06-19

**Authors:** Hayden P. Nix, Myles Sergeant, Nabha Shetty

**Affiliations:** 1 3688 Dalhousie University, Halifax, Nova Scotia, Canada; 2 3710 McMaster University, Hamilton, Ontario, Canada

## Abstract

Despite most Canadians preferring to die at home, over 50% die in hospitals, a setting often discordant with patient-centered end-of-life care and environmentally harmful. This article argues that healthcare policies unintentionally “nudge” patients and providers towards the medicalization of death, contributing to low-value care and significant greenhouse gas emissions. We analyze how inaccessibility to primary and palliative care, default “full code” status, overspecialization, and inadequate home-care supports perpetuate hospital deaths. Using an illustrative case, we demonstrate how these policies influence care trajectories from outpatient to hospital admission and disposition planning. Our aim is to highlight these underrecognized downstream effects to inform health leaders about opportunities to improve end-of-life care quality, align with patient preferences, and secondarily, benefit planetary health.

## Introduction

Medicalization is the process of labelling natural biological occurrences as problems that require medical intervention.^1^ In Canada, death is medicalized. After age 70, Canadians’ healthcare resource use increases exponentially, largely via hospital-based care.^2^ Over 60% of Canadians are admitted to hospital in their last year of life—most for over a month—resulting in over 5 million hospital bed-days annually.^3-5^ Ultimately, most Canadians die in hospital.^6^

Dying in hospital is often discordant with patient-centred care. Approximately 87% of Canadians prefer to receive end-of-life care and die at home and, when engaged in detailed care planning, most residents of Long-Term Care (LTC) facilities prefer to avoid hospitalization and receive only care in place.^7-10^ Dying in one’s preferred location is strongly associated with family member satisfaction with end-of-life care.^11^

Further, dying in hospital negatively impacts the environment. Hospitals are resource intensive settings, using high volumes of disposable goods alongside outsized energy, ventilation, and heating usage.^12,13^ Fingertip accessibility of investigations and interventions in hospital further promotes overuse. Annually, ward-level hospital care for Canadians in their last year of life produces 221,926-263,900 tonnes of Greenhouse Gas Emissions (GHGs).^2^

But if dying in hospital is so detrimental to patients, families, and the environment, then why is it so common? There is a growing body of literature surrounding the use and ethics of “nudging” in health-related decision-making.^14-18^ Nudging is defined as “any aspect of the choice architecture that alters people’s behaviour in a predictable way without forbidding any options or significantly changing their economic incentives”.^14^ Health-related system design, legislation, policies, and the way clinicians discuss medical issues subconsciously influence provider and patient behaviour. Understanding nudging in this context could help identify modifiable contributors to clinical outcomes, including the unintentional medicalization of death.

In this article, we argue that some healthcare policies nudge patients and healthcare teams unintentionally towards medicalizing death. We use an illustrative clinical case that outlines three stages that many patients experience near the end-of-life: outpatient care, hospital admission, and disposition planning to identify and analyze relevant policies. Our analysis aims to inform health leaders about the under-recognized, unintentional downstream effects of these healthcare policies that contribute to medicalizing death, thereby propagating low-value care driven greenhouse gas emissions. We recognize the complexity of these problems; providing comprehensive policy solutions is beyond the scope of this article. Further, there are circumstances where a medicalized death is consistent with a patient’s values and preferences. We do not argue that patients should be denied this option, nor that planetary health should be a consideration in end-of-life decision-making. Instead, we aim to demonstrate that there may be opportunities for health leaders to facilitate the provision of high-quality end-of-life care, and that doing so is beneficial to patients and, secondarily, the planet.

## Illustrative case: Part 1

Consider the following hypothetical case. Ms. M. is a 73-year-old living at home with her husband in rural Nova Scotia, near a pulp and paper mill. Her medical history includes chronic obstructive pulmonary disease, lyme disease, coronary artery disease, hypertension, dyslipidemia, hypothyroidism, and depression. She obtains medications from her respirologist, cardiologist, and walk-in clinics due to lack of a consistent primary care provider.

Last year, she was diagnosed with lung adenocarcinoma with spinal and liver metastases. Her ability to do heavy housework and grocery shopping were limited by back pain and shortness of breath. She received radiation therapy and started a tyrosine-kinase inhibitor. In conversations with her family, she consistently expressed preferences to “fight” her cancer for as long as she could, and to eventually die at home, surrounded by family.

Her treatment was complicated by nausea, diarrhoea, cough, and shortness of breath. Her frailty progressed to needing assistance walking from the bed to the bathroom. After attending her grandchild’s birthday party, she developed a sore throat and worsened shortness of breath. Unable to get out of bed, she called 911.

## Outpatient care

After receiving a terminal diagnosis, several healthcare policies nudge patients, families, and providers away from palliative care, towards more aggressive approaches for disease management. In this section, we outline how the inaccessibility of primary and palliative care nudges toward inappropriate medicalization that culminates in presentation to hospital.

### Primary care access blocks

An estimated 6.5 million Canadians—over 20% of the population—do not have a primary care provider.^19^ Nationally, there are approximately 20,000 family physician job openings.^20^ The number of medical students choosing to specialize in family medicine continues to decrease.^19^ Among those who train in family medicine, many practice in niche areas such as emergency medicine, hospitalist, or mental health, instead of primary care.^21^

Manifold policies nudge medical students away from primary care. Medical school admissions criteria reward graduate degrees, thereby favouring those interested in specialization.^22^ In addition, medical school curriculum glorifies specialization.^23^ Further, family physicians often work in private practice, in remuneration models that are underfunded compared with hospital-based specialists. Compensation levels fail to capture the requirements of the clinical and administrative complexity in effectively managing patients living with multimorbidity.^19,24^

Without a primary care provider, patients with terminal illnesses are nudged towards medicalization in at least two ways. First, they lack access to advanced care planning. Primary care providers are ideally situated to discuss goals of care with patients because of interpersonal continuity.^25^ Without this relationship, patients may lose the opportunity to discuss and document preferences pertaining to their goals of care and end-of-life care. For some patients, this may lead to unwanted aggressive management of terminal medical issues. In fact, lacking an advanced directive is associated with dying in hospital.^26^

Second, discontinuity of care nudges toward medicalization. Primary care providers are integral for care coordination. In their absence, outpatient subspeciality care becomes fragmented, inefficient, and redundant causing increased healthcare utilization, adverse events, emergency department visits, and hospital admissions.^25,27,28^

### Palliative care access blocks

The Canadian Society for Palliative Care Physicians estimates that Canada needs double the number of palliative care physicians.^29^ Only 20% of Canadians living with terminal illness receive publicly funded palliative home care in their last year of life.^30^ Among Canadians who receive palliative care, 50% obtain access in their last 22 days.^31^ Palliative care physician shortages are an extension of primary care shortages; over 90% of physicians who provide palliative care are family physicians.^32^

Like family medicine, policies nudge providers away from choosing palliative medicine as a speciality. Palliative medicine is not prioritized in medical training, with most students receiving fewer than 10 hours of exposure during medical school.^29^ Several Canadian medical schools lack a division of palliative medicine or residency positions.^33^ Furthermore, creation of the 5-year Royal College subspeciality residency has extended training and disincentivized those favouring a mixed generalist practice.^34^ These systemic deficits in clinical expertise are compounded by shortages of Canadian home care services, thus making home palliation an impossibility for most Canadians.

The disproportionate accessibility of hospital-based care over primary care and palliative care nudges patients with terminal illnesses to present to acute care settings when their condition worsens. Access to outpatient palliative care reduces emergency department visits, hospital, and Intensive Care Unit (ICU) admissions suggesting palliative care empowers death at home.^35^ Further, one study found that 58% of patients presenting to the emergency department did so because they could not access care elsewhere.^36^

## Illustrative case: Part 2

In the emergency department, Ms. M. was found to have progressive cancer with a pleural effusion. She was admitted to internal medicine, and during this process was asked if she wanted “everything done.” She answered affirmatively, and “full code” status was documented. The internal medicine team consulted medical oncology, respirology, and gastroenterology while ordering an array of investigations. Diagnostic conclusions were that cancer progression with a malignant pleural effusion, medication toxicity, and a viral infection drove her functional decline. An indwelling pleural catheter was inserted, and her respiratory symptoms improved. However, Ms. M. subsequently acquired influenza A and *Clostridium difficile* infections while in hospital.

## Hospital admission

Once a patient with a terminal illness presents to the emergency department, many policies nudge toward aggressive care. In this section, we demonstrate how (1) policies that make “full code” the default code status, and (2) policies that incentivize subspecialized in-patient care contribute to medicalizing death.

### Full code default

In Canada, hospital policies that govern goals of care default to “full code” status in the absence of documented goals of care conversations. This means, regardless of clinical context, patients may receive life sustaining treatment, including Cardiopulmonary Resuscitation (CPR), mechanical ventilation, and intensive care unit admission.

A “full code” default nudges providers and patients to medicalize death. Generally, default options are often interpreted as recommendations.^37^ Thus, a “full code” default nudges providers, particularly trainees, to offer “everything” and nudges patients to accept or request it. However, even mild frailty is strongly associated with reduced odds of surviving CPR and surviving to discharge from hospital after CPR.^38^ It may be especially difficult for inexperienced healthcare providers to overcome this nudge because conducting goals of care discussions is a complex clinical skill. A successful goal of care discussion is one that aligns the patient’s code status with their values, preferences, and illness trajectory. Unfortunately, Canadian medical trainees receive insufficient training on goals of care discussions.^39^ Lacking expertise on when and how to recommend different code statuses leaves physicians vulnerable to be nudged towards the “full code” default.

Even if patients do not end up requiring CPR or ventilation, a “full code” designation nudges towards more aggressive approaches to care. Other code status designations indicate that some investigations and treatments may be too invasive, risky, or uncomfortable for the patient. This prompts healthcare teams towards a more holistic approach to care because they must weigh patients’ values, preferences, and comfort when creating care plans. By contrast, “full code” designation removes this prompt and nudges healthcare teams towards providing reflexive care for the patient’s active issues without considering the patient’s overall quality of life or illness trajectory.

### Overspecialization

Modern medicine is increasingly specialized.^40-43^ Specialization nudges toward overtesting and overtreatment in patients living with frailty or terminal illness. Most specialists are trained and remunerated to investigate and intervene upon specific organ systems. Compensation models disincentivize conservative care by rewarding procedures and acute interventions thereby nudging healthcare teams to medicalize death. Recognizing when a patient is at the end-of-life requires a holistic perspective to understand how medical issues, investigations, and treatments impact a patient’s function, quality of life, and illness trajectory. Furthermore, communication about prognosis and navigating complex decision-making is a skill in and of itself not ascribed to a particular speciality.^44^ By contrast, the subspecialist perspective is, in principle, reductionist: “oriented toward or organized around body parts instead of treating the whole patient in their life context.”^43^ Overspecialized in-patient care is associated with increased number and invasiveness of investigations, prolonged length of stay, and adverse outcomes.^40,42,45,46^ Like “full code status,” involvement of multiple subspeciality services nudges patients and providers towards attempting to fix specific issues, while overlooking the big picture that care approaches grounded in generalism bring.

## Illustrative case: Part 3

During her admission, Ms. M. was too unwell to work with physiotherapy. She required assistance for personal care and became bed-bound due to back pain. Ms. M.'s daughter could not take time off work to support a discharge home; thus, Ms. M. was designated as “Alternate Level of Care” (ALC) and placed on waitlists for hospice and LTC facilities. She died in hospital awaiting transfer to another facility.

## Disposition planning

Once end-of-life patients are admitted to hospital, several policies keep them there. In Canada, approximately 17% of acute care beds are occupied by ALC patients,^47^ limiting the availability of acute care beds. Nearly 40% of patients deemed ALC are in their last 90 days of life,^48^ and half die in hospital awaiting transfer to another location.^49^ Below, we outline how inaccessible home care nudges patients to remain in hospital at the end-of-life.

### Home care access blocks

Home care is not included in the Canada Health Act and has long suffered from underfunding and inadequate oversight.^50^ Nationally, there is a marked shortage in home care workers, and demand is projected to continue to increase.^51,52^ Only some home and community care supports are publicly funded, meaning that many older adults cannot afford to access these services.^53^ Some patients may have family or friends who are willing and capable of providing end-of-life care at home. However, this may be financially challenging. Caregiver benefits (i.e., funding for family members or friends to act as caregivers for patients living with frailty) are difficult to access and do not provide a livable wage.^54,55^

The inaccessibility of home care nudges patients to a medicalized death. Quite simply, without a way to return home safely, patients opt to remain in hospital and await transfer to another institution, such as LTC or hospice. Due to shortages in LTC and hospice beds, patients often end up remaining in hospital until they die.

## Discussion

Even though 87% of Canadians prefer to receive end-of-life care at home, over 50% of Canadians die in hospital. Manifold policies nudge patients, families, and healthcare teams towards dying in hospital. The Canadian healthcare system and medical education are structured to prioritize acute care over primary care, palliative care, and home care. The fact that acute care is somewhat more accessible than primary care, palliative care, and home care for some, particularly in urban parts of the country, nudges many patients with terminal illnesses to seek help at hospitals. Once a patient presents to hospital, policies that govern code status and models of care nudge patients and healthcare teams towards aggressive approaches to care. And once a patient is admitted to hospital, policies that fail to support robust home care nudge patients to remain in hospital long after their acute medical issues have resolved, often until they die in hospital.

**Figure 1.**
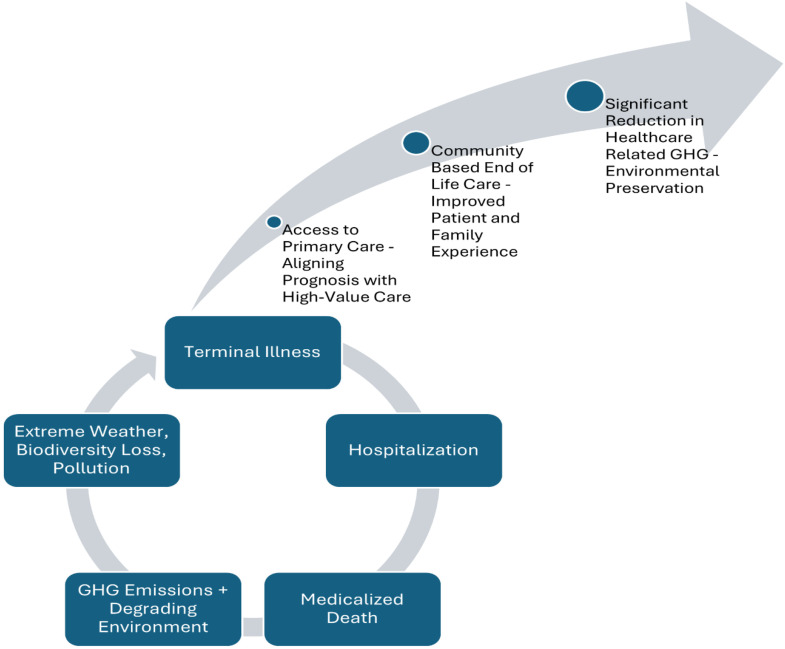
Investment in primary care and community-based end-of-life care will reduce the medicalization and environmental impact of end-of-life care.

The environmental impact of dying in hospital is substantial and unnecessary. In Canada, one ward bed-day in hospital produces an estimated 23 kg CO_2_e, not including the impact of the supply chain.^2^ By contrast, transferring to a home care setting decreases daily emissions to an estimated 1.3 kg CO_2_e.^2^ One day in intensive care leads to one-hundred-fold greater emissions. From 2021 to 2022, 44,000 Canadians were admitted to hospital for palliative care.^31^ The average length of stay was 12 days, adding up to a cumulative 528,000 bed-days in hospital.^31^ If the 87% of these patients who wish to die at home were empowered to do so, not only would it align with their choice, it would also reduce emissions produced by end-of-life care by nearly 10 million kg CO_2_e per year. Notably, the current environmental impact of home care is likely underestimated secondary to a lack of resources allocated to the sector. If more resources (e.g., equipment, single use medical devices, and personnel) are allocated to the home care sector, the environmental impact will increase. However, even if home care were well-resourced, its environmental impact is likely to remain lower than hospital-based care. Ironically, the health impacts of the climate crisis affect vulnerable and equity deserving populations disproportionately, the same groups that often lack access to primary care. Thus, the exit strategy for this cycle of climate-perpetuated human illness that results in wasteful, high-intensity end-of-life care, which further degrades population and planetary health, is to invest in upstream primary care interventions and support community-based end-of-life care (Figure 1).

## Conclusion

Dying in hospital is often discordant with both patient-centred care and environmental sustainability. In this article, we have identified manifold policies that nudge patients, families, and healthcare teams towards the unintentional medicalization of death. Health leaders need to look for opportunities to modify or mitigate the hidden consequences of these policies and allocate resources to promote community-based care. Doing so will simultaneously reduce unnecessary environmental harm and improve the quality of care provided to patients and families at the end-of-life.
